# Percutaneous Versus Open Management of Thoracic and Lumbar Hyperostotic Fractures: A Case-Control Study

**DOI:** 10.7759/cureus.94737

**Published:** 2025-10-16

**Authors:** Mark Lawlor, Mina Botros, Clarke Cady-McCrea, Paul T Rubery, Emmanuel N Menga, Robert Molinari, Mark Ehioghae, Lancelot A Benn, Kevin Yoon, Addisu Mesfin

**Affiliations:** 1 Orthopaedics, Massachusetts General/Brigham and Women’s Hospital, Boston, USA; 2 Orthopaedic Surgery, University of Rochester Medical Center, Rochester, USA; 3 Orthopaedic Surgery, University of Rochester, Rochester, USA; 4 Orthopaedic Spine Surgery, University of Rochester, Rochester, USA; 5 Orthopaedics and Rehabilitation, University of Rochester Medical Center, Rochester, USA; 6 Orthopaedic Surgery, MedStar Orthopaedic Institute, Washington, DC, USA; 7 College of Medicine, University of Rochester School of Medicine and Dentistry, Rochester, USA

**Keywords:** ankylosing spondylitis, diffuse idiopathic skeletal hyperostosis, dish, hyperostotic spine, minimally invasive, spinal cord injury, spine fractures, spine trauma

## Abstract

Background

This study aims to examine the surgical outcomes and complications associated with minimally invasive stabilization using percutaneous pedicle screws (MIS-PPS) versus open surgical management (OSM) among patients with spine fractures in the setting of hyperostotic spine diagnosis.

Methodology

This retrospective, case-control study was conducted at a single Level I Trauma Center, including 30 consecutive patients (nine ankylosing spondylitis patients and 21 diffuse idiopathic skeletal hyperostosis patients) who underwent surgery for thoracolumbar extension-distraction fractures. Collected data included patient demographics, comorbidities, injury mechanism, spine region, fracture level, and intraoperative and postoperative complications. Continuous variables included age, body mass index, estimated blood loss, operative time, number of levels instrumented, and length of stay.

Results

Between 2002 and 2020, 15 patients underwent MIS-PPS (3 females, 12 males, average age of 75.3 years) and 15 underwent OSM (3 females, 12 males, average age of 73.6 years). The MIS-PPS group had significantly lower blood loss (95 ± 31.6 mL vs. 643.3 ± 534.4 mL; p < 0.001) and shorter operative time (130.7 ± 36.4 minutes vs. 208.7 ± 41.8 minutes; p < 0.001). They also required fewer levels of instrumentation (5.2 vs. 6.8; p < 0.001). There were no significant differences in postoperative infection rates, epidural hematomas, or implant revisions between the groups.

Conclusions

MIS-PPS provides the benefits of reduced blood loss, shorter operative time, and fewer levels of instrumentation compared to OSM, with no significant differences in postoperative complications.

## Introduction

Ankylosing spondylitis (AS) is a chronic inflammatory condition characterized by progressive endochondral ossification of the cartilage followed by joint destruction and ankylosis, as well as ossification of the disc, referred to as syndesmophytosis. It is the most common seronegative spondyloarthropathy, with a prevalence of 0.1% to 1.4%, occurring predominantly in males compared to females (3:1 ratio) [1]. Past literature has suggested that AS may have a geographic association, especially in predominantly Caucasian countries, such as Norway [2-5]. The high prevalence in these populations has been linked to the HLA-B27 allele, which was found in 90-95% of AS cases in white patients [6]. AS progression has been described to proceed in a caudal-to-cranial fashion, typically starting from the sacroiliac joints and ascending the cervical spine [7]. From a biomechanical standpoint, ossification across multiple vertebrae lengthens the lever arm of the spine, and in combination with osteoporosis, increases the risk of spinal fractures from falls or low-energy trauma [8,9]. Likewise, diffuse idiopathic skeletal hyperostosis (DISH) is characterized by chronic ossification and calcification of ligaments and tendons that lead to similar biomechanical changes as AS and elevated risk of vertebral fracture [10]. However, the pathophysiology is not well understood.

Together, DISH and AS are the primary causes of hyperostotic spine, a combination of back pain and spinal stiffness [11]. Patients diagnosed with a hyperostotic spine have four to five times higher risk of sustaining a low-energy unstable spinal column injury compared to patients without spine pathology [11]. Due to the brittle nature of hyperostotic bone, patients are also at a much higher risk of spinal cord injury secondary to spinal fractures. Overall, 75% of fractures occur in the cervicothoracic region, but up to 14% can be in the lumbar spine. Unfortunately, many of the hyperostotic spine disease fractures are seen with advanced imaging; as a result, these patients tend to have an increased rate of mortality and neurological deficits [12].

Non-operative management plays a small role in hyperostotic spinal fractures. Patients treated non-surgically tend to have a higher risk of complications, including decubitus ulcers, thromboembolism, and pulmonary complications [13]. Therefore, surgical management is preferred [14,15]. However, a hyperostotic spine can change the curvature of the spine, creating additional challenges operatively. For instance, patients with AS develop kyphosis of the spine due to a chronic adaptation to unload facet joints of pressure and pain [16]. One potential way to lessen the burden on both the patient and surgeon is treatment using minimally invasive stabilization and percutaneous pedicle screws (MIS-PPS). Historically, MIS-PPS has been shown to decrease operative time and blood loss compared to traditional open surgical management (OSM). However, there is a paucity of literature comparing techniques, especially in the case of hyperostotic vertebral fractures. Thus, this study aims to examine the surgical outcomes and complications associated with MIS-PPS versus OSM among patients with spinal fractures in the setting of hyperostotic spine disease.

## Materials and methods

Patient sample

**Figure 1:**
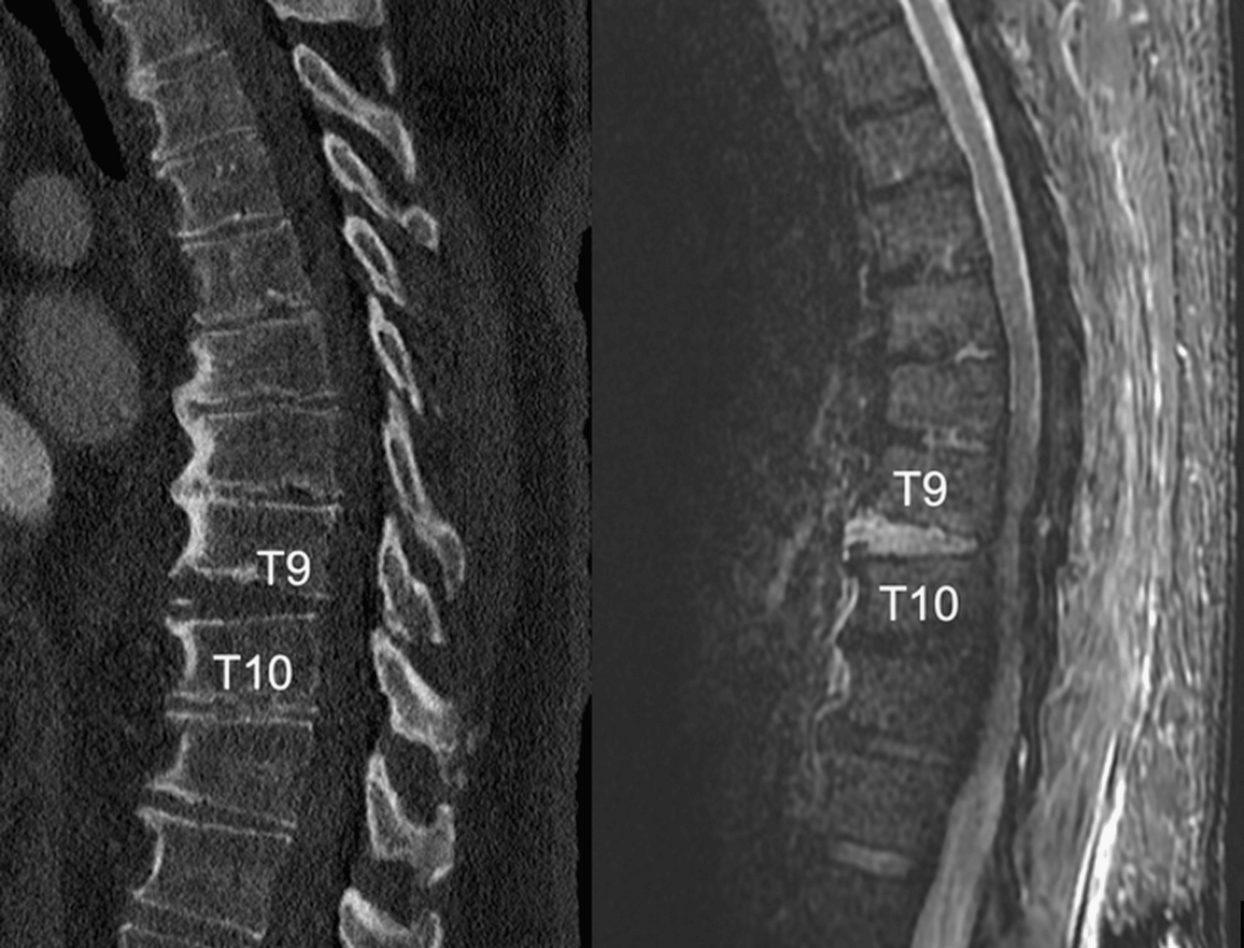
A 72-year-old male with DISH and a T9-T10 hyperextension fracture seen on CT and MRI. DISH = diffuse idiopathic skeletal hyperostosis

**Figure 2:**
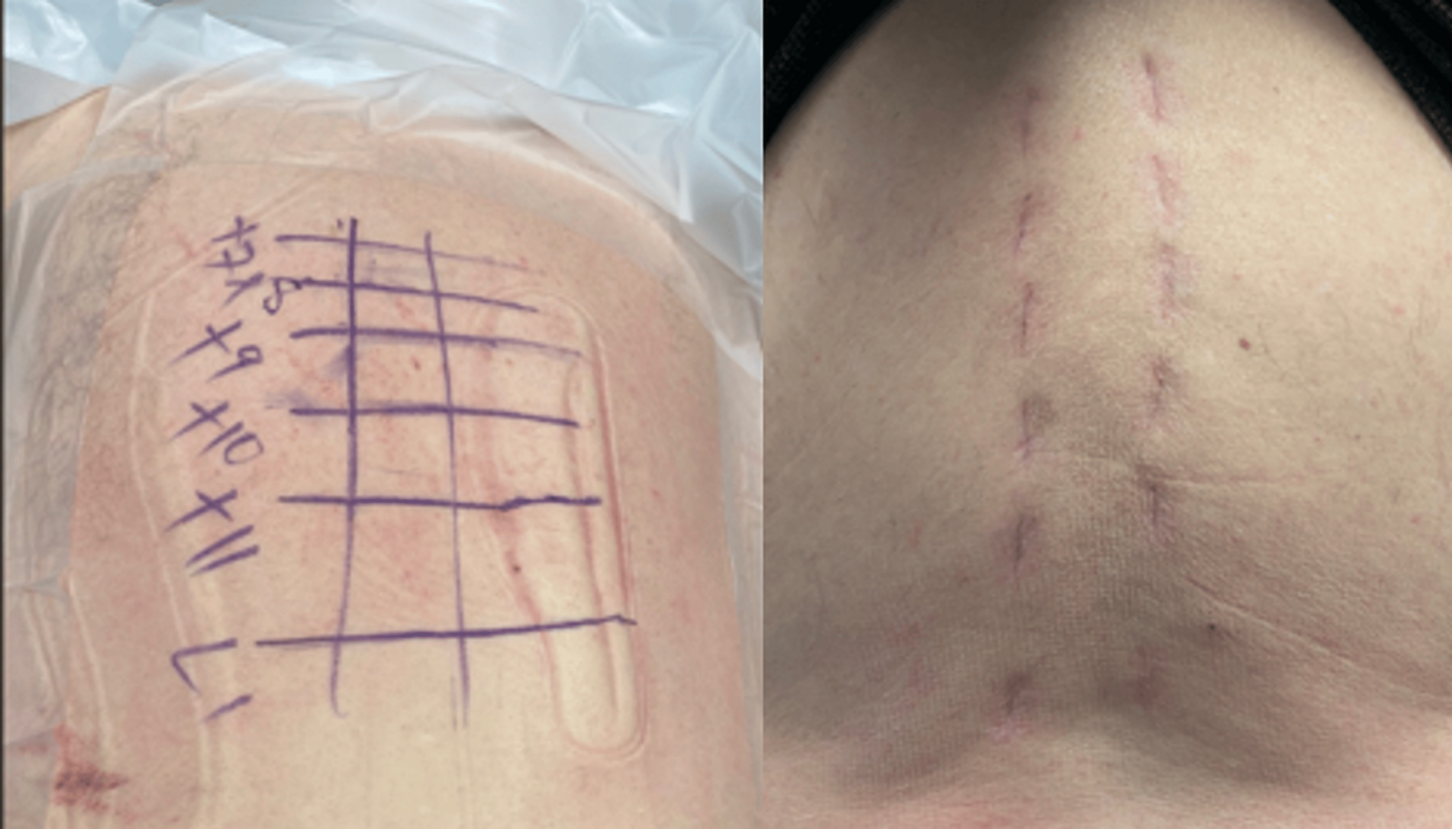
Intraoperative skin markings and incisions from the MIS-PPS instrumentation. MIS-PPS = minimally invasive stabilization-percutaneous pedicle screws

**Figure 3:**
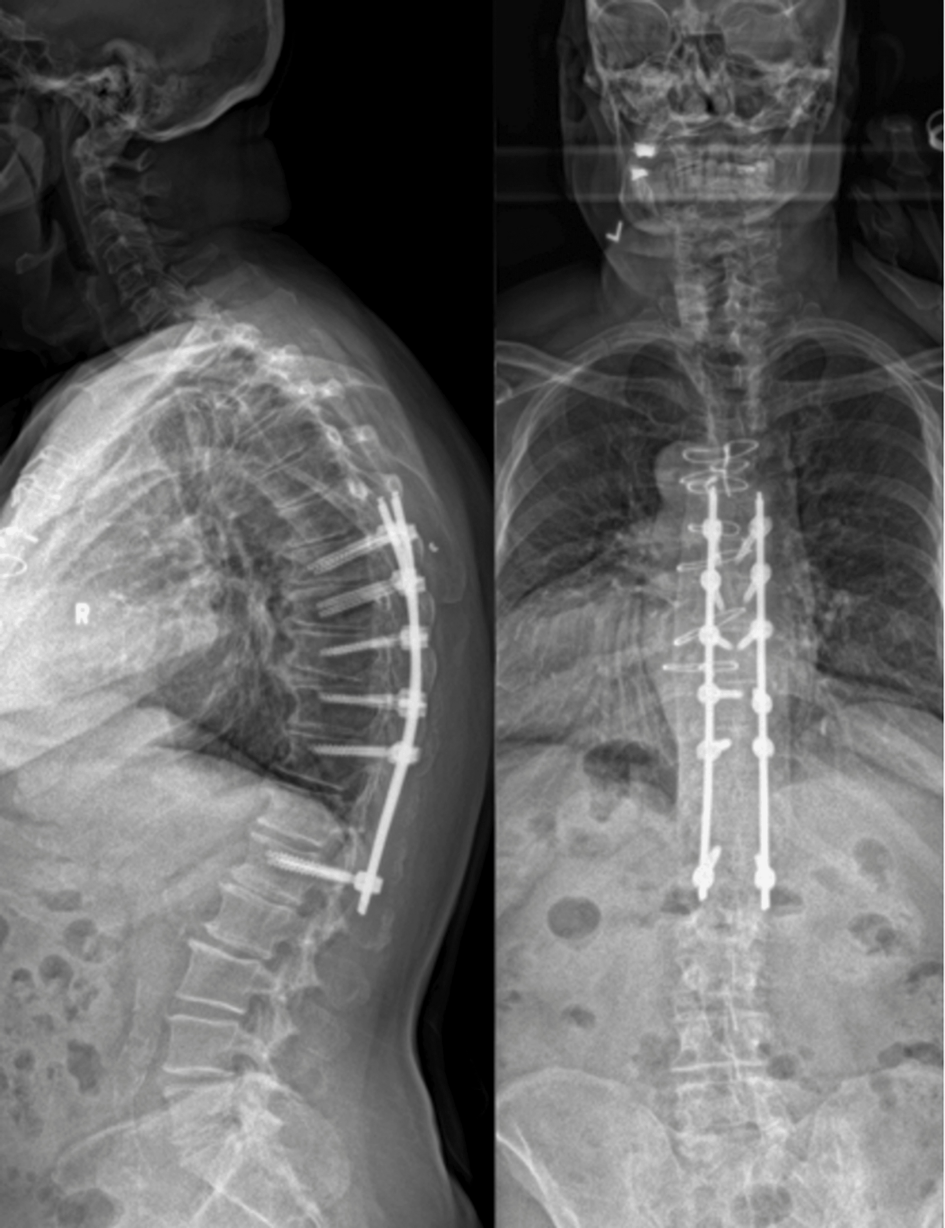
Postoperative AP/lateral radiographs at the sixth-month outpatient visit after undergoing MIS-PPS instrumentation. AP = anterior-posterior; MIS-PPS = minimally invasive stabilization-percutaneous pedicle screws

This retrospective study analyzed 30 consecutive patients with a history of AS or DISH admitted to a Level 1 Trauma Center (academic institution) between 2002 and 2020. Inclusion criteria included those who were older than 18 years old, were diagnosed with a thoracolumbar extension-distraction fracture with either AS or DISH, as well as a diagnosis of either AS or DISH determined radiographically. Exclusion criteria included a history of prior surgery of the hyperostotic spinal region, spinal malignancy, and a history of prior fracture. The cohort was composed of two groups, namely, MIS-PPS (Figures 1-3) and OSM. Patients in both groups were matched based on age and sex. A cohort of 15 patients who underwent MIS-PPS was matched to a cohort of 15 patients who underwent OSM during the same time period.

Categorical variables recorded included race (white, black, or other), sex (male or female), pre-existing comorbidities listed in the electronic medical record, mechanism of spine injury (ground-level fall, fall from a standing height, motor vehicle collision, low-energy trauma, and other), spine region and fracture level (cervical, cervicothoracic, thoracic, thoracolumbar, lumbar, and lumbosacral), presence of spinal cord injury (yes or no), American Spinal injury Association grade, and intraoperative and postoperative complications (implants revision, infection, and epidural hematoma). Continuous variables recorded included age, body mass index (BMI), estimated blood loss (EBL), operative time, number of levels instrumented, length of stay, and mortality (in months, calculated by the difference between the surgical discharge date and the deceased date).

Statistical analysis

Descriptive variables were reported. Bivariate analysis was performed to evaluate the following: (I) patients undergoing MIS-PPS versus patients undergoing OSM; (II) the continuous characteristics of the age and BMI were compared between both surgical techniques; (III) the EBL, operative time, length of stay, and number of levels instrumented were compared between MIS-PPS and OSM; (IV) the rate of reoperation, implant revision, infection, and epidural hematoma were compared between both surgical techniques; and (V) characteristics of the patient, mechanism of injury, spine fracture location were compared for patients who underwent MIS-PPS and OSM. The chi-square test was used to examine categorical variables between the two surgical techniques. In instances where categorical variables were reported on fewer than five, Fisher’s exact test was utilized. For continuous variables, a t-test was used. Multivariate logistic regression models were used to compare EBL, operative time, number of levels instrumented, and length of stay. Statistical significance was set at a p-value <0.05.

## Results

Patient sample characteristics

From 2002 to 2020, data on 15 patients undergoing MIS-PPS were collected. The group was composed of three females and 12 males with an average age of 75.3 years (range = 51-86 years). All patients in this cohort identified their race as white. Additionally, these patients had greater than six (40%) comorbidities, and an average BMI of 32.8 kg/m² (±4.39). Ten patients who underwent MIS-PPS had been diagnosed with DISH, and the remaining five had been diagnosed with AS. The most prevalent insurance type for this surgery was Medicare (n = 8, 53.3%), followed by private insurance (5, 33.3%), and Medicaid (2, 13.3%). The majority of patients undergoing MIS-PPS had sustained a ground-level fall (8, 53.3%), followed by a fall from height (2, 13.3%) and a motor vehicle collision (4, 26.4%). Overall, 93.3% of injuries occurred below T6 (Table 1).

**Table 1:** Demographic analysis of patients diagnosed with AS and DISH. ASIA = American Spinal Injury Association Impairment; BMI = body mass index; SD = standard deviation; DISH = diffuse idiopathic skeletal hyperostosis; AS = ankylosing spondylitis

Characteristic	AS (n = 15)	DISH (n = 15)	P-value	Statistic test
Age, years; mean ± SD	73.6 ± 6.6	75.3 ± 12.0	0.341	t = 0.97
Sex, n (%)				
Male	11 (73.3)	12 (80.0)	0.59	χ² = 0.29
Female	4 (26.7)	3 (20.0)		
Race n (%)				
White	14 (93.3)	12 (80.0)	0.405	χ² = 1.81
Black	1 (6.7)	1 (6.7)		
Other	0 (0)	2 (13.3)		
BMI, mean (range)	33.2 ± 3.85	32.2 ± 7.09	0.625	t = 0.49
BMI (n (%))				
Underweight (<18.50 kg/m^2^)	0 (0)	0 (0)	0.333	χ² = 3.41
Normal (18.50-24.99 kg/m^2^)	0 (0)	2 (13.3)		
Overweight (>25 kg/m^2^ and <29.99 kg/m^2^	4 (26.7)	4 (26.7)		
Obese (≥30 kg/m^2^)	11 (73.3)	9 (60)		
Comorbidities, n (%)				
<5	10 (66.7)	11 (73.3)	0.69	χ² = 0.16
≥5	5 (33.3)	4 (26.7)		
Cause of spine fracture, n (%)				
Ground-level fall	4 (26.7)	7 (46.7)	0.558	χ² = 1.17
Fall from height	2 (13.4)	1 (6.7)		
Motor vehicle collision	9 (60.0)	7 (46.6)		
Spine level(s) fractured, n (%)				
Lower cervical	0 (0)	3 (20.0)	0.134	χ² = 5.58
Upper thoracic	2 (13.3)	0 (0)		
Lower thoracic	11 (73.3)	12 (80.0)		
Lumbar	2 (13.3)	0 (0)		
Presenting ASIA grade, n (%)				
A	2 (13.3)	1 (6.7)	0.512	χ² = 3.28
B	0 (0)	1 (6.7)		
C	0 (0)	0 (0)		
D	0 (0)	0 (0)		
E	13 (86.7)	13 (86.7)		
Discharge destination				
Home	0 (0)	5 (33.3)	0.014	χ² = 3.28
Rehabilitation facility	15 (100)	10 (66.7)		

From 2002 to 2020, data on 15 patients undergoing OSM were collected. The group was composed of three females and 12 males, with an average age of 73.6 years (range = 49-90 years). Overall, 80% of patients in the cohort were white. The average BMI within this group was 32.63 kg/m² (±6.8). In total, 11 patients had DISH, and four had AS. The most prevalent insurance type was Medicare (10, 66.6%), followed by private insurance (4, 26%), and Medicaid (1, 6.6%). The most common mechanism of injury was a ground-level fall (12, 80%), followed by a fall from height (2, 13.3%), and a motor vehicle collision (2, 13.3%). Overall, 66% of injuries occurred below T6 (Table 1).

In this study, patients diagnosed with AS had a 100% disposition to a rehabilitation facility on discharge. In comparison, among patients diagnosed with DISH, 66.7% were discharged to a rehabilitation facility (p = 0.018).

Surgical technique

For the MIS-PPS, fracture distribution was as follows: T7-T8, n = 3; T9-T10, n = 4; and n = 1 each at T5, T8, T9, T10, T10-T11, T11, T11-T12, and L1-L2. For the OSM group, fracture distribution was as follows: T6, n = 2; T9, n = 2; T11, n = 2; and n = 1 each at T5, T6-T7, T7-T8, T9-T10, T10, T10-T11, and T11. One patient in the MIS-PPS and two patients in the OSM group had surgery for cervical spine fractures.

A comparison of the surgical outcomes between the MIS-PPS and OSM groups is presented in Table 2. The MIS-PPS group demonstrated less operative blood loss (95 ± 31.6 mL) compared to the OSM group (643.3 ± 534.4 mL) (p < 0.001). Additionally, patients undergoing MIS-PPS had a shorter operative time (130.7 ± 36.4 minutes) compared to the OSM group (208.7 ± 41.8 minutes) (p < 0.001). Those undergoing MIS-PPS had shorter fixation vertebral lengths (5.2; range = 5-8) compared to the OSM group (6.8; range = 4-10) (p < 0.001) (Table 3).

**Table 2:** Comparison of the surgical outcomes between the OSM and MIS-PPS surgical management groups. BMI = body mass index; MIS-PPS = minimally invasive stabilization-percutaneous pedicle Screw; OSM = open surgical management

Characteristic	Open (n = 15)	MIS (n = 15)	P-value	Statistical test
Age, years, mean (range)	73.6 (49-90)	75.33 (51-86)	0.628	t = 0.49
Sex, n (%)				
Male	11 (73.3)	12 (80)	0.666	χ² = 0.19
Female	4 (26.7)	3 (20)		
Race, n (%)				
White	11 (73.3)	15 (100)	0.325	χ² = 2.25
Black	2 (13.3)	0 (0)		
Other	2 (13.3)	0 (0)		
Insurance type, n (%)				
Private	4 (26%)	5 (33.3)	0.057	χ² = 5.73
Medicare	10 (66.6%)	8 (53.3)		
Medicaid	1 (6.6%)	2 (13.3)		
BMI, mean (SD)	32.63 (6.81)	32.81 (4.39)	0.413	t = 0.83
BMI, n (%)				
Underweight (<18.50 kg/m^2^)	0(0)	0(0)	0.247	χ² = 2.80
Normal (18.50–24.99 kg/m^2^)	1 (6.7)	1 (6.7)		
Overweight (>25 kg/m^2^ and <29.99 kg/m^2^	6 (40.0)	2 (13.3)		
Obese (≥30 kg/m^2^)	8 (53.3)	12 (80.0)		
Comorbidities, n (%)				
<5	12 (80)	9 (60)	0.232	χ² = 1.43
≥5	3 (20)	6 (40)		
Cause of spine fracture, n (%)				
Ground-level fall	5 (33.3)	6 (40.0)	0.779	χ² = 0.50
Fall from height	2 (13.3)	1 (6.7)		
Motor vehicle collision	8 (53.3)	8 (53.3)		
Spine level(s) fractured, n (%)				
Lower cervical	2 (13.3)	1 (6.7)	0.088	χ² = 6.54
Upper thoracic	2 (13.3)	0 (0)		
Lower thoracic	9 (60.0)	14 (93.3)		
Lumbar	2 (13.3)	0 (0)		
Total blood loss (mL), mean (SD)	643.3 (534.4)	95 (31.65)	<0.001	t > 3.67
Operative time (minutes), mean (SD)	117.5 (27.8)	130.6 (9.1)	0.2915	t = 1.08
Length of hospital stay, mean (SD)	13.2 (7.03)	12.2 (8.5)	0.654	t = 0.45
Levels fused, n (range)	6.15 (4–10)	5.2 (5–8)	0.012	t = 2.69
Discharge destination				
Home	1 (6.67)	4 (26.6)	0.142	χ² = 2.16
Rehabilitation facility	14 (93.3)	11 (73.4)		

**Table 3:** Comparison of patient complications by surgical technique. Regression models. OR = odds ratio; 95% CI = 95% confidence interval

	OR (95% CI)	P-value
Estimated blood loss (mL)	1.03 (1.01-1.06)	0.045*
Operative time (minutes)	0.99 (0.973-1.02)	0.584
Length of hospital stay (days)	1.03 (0.92-1.16)	0.580
Levels fused	2.24 (0.81-6.18)	0.119
Mortality (months)	1.13 (0.94-1.38)	0.193

Complications

There was one intraoperative complication in the MIS-PPS group: cardiac arrest secondary to a mucous plug. Postoperative infection rates were not significantly different between the two groups (one infection in the MIS-PPS group, and one infection in the OSM group) (p = 0.101). Two cases of wound complications occurred within 90 days postoperatively in the MIS-PPS group and were addressed non-operatively. In the OSM group, postoperative deep vein thrombosis occurred in two patients, and postoperative neurologic complications occurred in two patients (one foot drop and one neurogenic bowel/bladder with subsequent return to baseline). Neither group had any incidence of epidural hematoma or implant revision.

A comparison of patient complication rates by surgical technique is described in Table 4. There was only one case in each group requiring reoperation. There was no statistical difference in mortality within one year between the two surgical techniques (p = 0.921). However, there was a statistically significant difference in the mortality at one year based on the cause of hyperostotic spine pathology (p = 0.036) (Table 5).

**Table 4:** Comparison of patient complications by surgical technique. Fisher’s exact test performed; no corresponding raw statistical value. MIS = minimally invasive stabilization

Outcome, n (%)	Open (n = 15)	MIS (n = 15)	P-value
Reoperation	1 (6.7)	1 (6.7)	-
Mortality (within one year postoperatively)	2 (13.3)	3 (20)	0.921

**Table 5:** Comparison of patient complications by diagnosis. Fisher’s exact test performed; no corresponding raw statistical value. OR = odds ratio; DISH = diffuse idiopathic skeletal hyperostosis; AS = ankylosing spondylitis

Outcome, n (%)	AS (n = 15)	DISH (n = 15)	P-value
Reoperation	1 (6.7)	1 (6.7)	
Mortality (within one year postoperatively)	1 (16.7)	4 (26.7)	0.036

**Figure 4:**
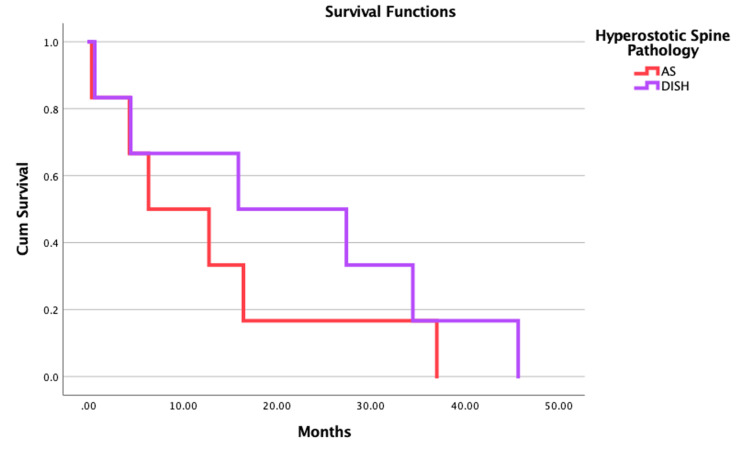
Kaplan-Meier curve demonstrating the survival function analysis based on the hyperostotic spine pathology (AS vs. DISH) (A) and surgical technique (open vs. MIS) (B) DISH = diffuse idiopathic skeletal hyperostosis; AS = ankylosing spondylitis; MIS = minimally invasive stabilization

**Figure 5:**
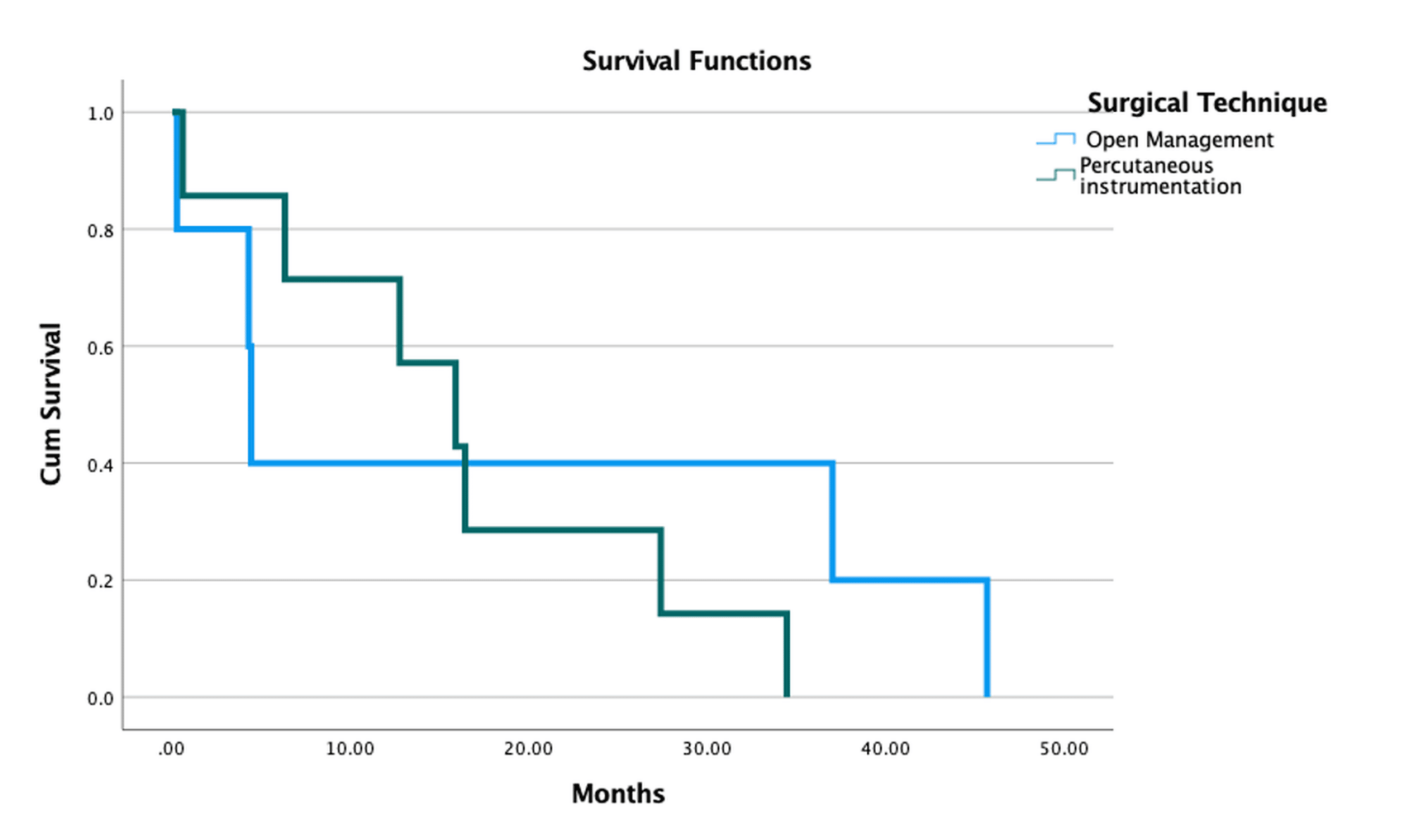
Kaplan-Meier curve demonstrating the survival function analysis based on the surgical technique (open vs. MIS). MIS = minimally invasive stabilization

Figure 4 depicts the Kaplan-Meier curve, indicating that there was no survival difference between patients with AS and those with DISH. Patients diagnosed with AS had a survival rate of 65% at five months and 42% at one year. Patients diagnosed with DISH had a survival rate of 65% at five months and 46% at one year. Additionally, Figure 5 depicts the Kaplan-Meier curve, indicating that there was no survival difference between patients who underwent open or percutaneous surgical procedures.

## Discussion

Both AS and DISH can cause hyperostosis spine disease and can present with spinal stiffness, osteoporosis, and a high risk of falls secondary to balance and gaze difficulties [17]. Patients with hyperostosis spine disease are more likely to sustain thoracolumbar extension-distraction fractures, likely due to the extended spinal lever arm, elevated rates of osteoporosis, and high incidence of ground-level falls [8,9,18,19]. The hyperostotic spine is inherently unstable, and fractures benefit significantly from surgical intervention [20-23]. The study examined the surgical outcomes and complications associated with MIS-PPS and OSM among patients with spinal fractures in the setting of hyperostotic spine disease.

Our findings indicate that surgical management of hyperostotic spine fractures of the thoracic/thoracolumbar spine can either be performed by MIS-PPS versus OSM, at the discretion of the spine surgeon. The percutaneous group (also known as the MIS-PPS group) had significantly fewer levels instrumented (5.2 levels) compared to the OSM group (6.8 levels). This finding is similar to previous studies reporting shorter segments with percutaneous compared to OSM [24-26].

Additionally, patients undergoing percutaneous instrumentation tend to have less EBL (95.5 mL; range = 50-150 mL) compared to the open group (495 mL; range = 100-900 mL). Similar findings have been reported by Hong et al. [27]. As one would expect, open incisions inevitably result in increased blood loss due to the amount of tissue disrupted compared to their minimally invasive counterparts, aligning with past research.

We found no significant difference in postoperative infections between the MIS-PPS and open surgical groups. This was unexpected, as one of the benefits of minimally invasive surgery is that there is less opportunity for skin flora or bacteria to enter the body. Results may be underreported due to sample size, and future studies with larger cohorts may help formulate a clearer understanding. In our study, no patient developed an epidural hematoma or needed an implant revision. Further, our study found no difference in the patient demographics or fracture characteristics based on whether the patient was diagnosed with AS versus DISH. Similar findings have also been reported by Bernstein et al. and Teunissen et al. [17,28]. This is expected as the pathologies are very similar in presentation, symptoms, and anatomic changes.

Multiple studies have reported shorter operative times (Kruger et al.: 60.2 minutes; range = 32-135 minutes; Nayak et al.: 227 minutes; range = 79-449 minutes), less blood loss (Nayak et al.: 251 mL; range = 25-900 mL), and good functional outcomes [18,29,30]. Moussallem et al. compared OSM and MIS-PPS, showing similar results as our findings in terms of operative time (MIS-PPS: 254.8 minutes vs. open: 334.7 minutes), less blood loss (MIS-PPS: 166.8 mL vs. open: 1,240.5 mL), shorter hospital stays (MIS-PPS: 9.6 days vs. open: 16.5 days), lower complication rates (MIS-PPS: 56% vs. open: 87%), and good functional outcomes [18,29,30]. Lindtner et al. also compared percutaneous and open surgical techniques to address surgical fractures and hyperostotic spine disease. They reported that those who underwent the traditional OSM had higher postoperative complication rates (1.3% vs. 0.7%) [31]. Bredin et al. performed a similar comparison to our study and reported that both surgical techniques were associated with improvement in postoperative pain [26].

Our study has several limitations. Foremost, the retrospective nature of our study has its inherent bias. It is also important to note that all patients were from a single urban, Level 1 Trauma, academic medical center. Therefore, it may be difficult to generalize the findings of the study to other dissimilar healthcare centers. There may be selection bias, as the surgical technique (MIS-PPS vs. OSM) was determined by the attending spine surgeon at admission and evaluation. Further, the sample size in the study was small due to the low prevalence of stiff spine fractures in the setting of hyperostotic spine disease; several previous studies evaluating hyperostotic spine disease reported a similar sample size [27]. Therefore, a prospective study is warranted to better examine the true difference between MIS-PPS surgery and the traditional OSM and treating spinal fractures among patients with hyperostotic spine disease. Additionally, a high mortality rate (odds ratio = 1.13, n = 5) was found in the study, although insignificant (p = 0.193). Inherently, the average age of the cohort (73 and 75 years, respectively, for open and MIS-PPS groups), high BMI, and the proportion of subjects with multiple comorbidities put them at risk for mortality secondary to non-surgical causes. Future studies with a greater distribution of younger patients may help provide greater significance to the mortality trends postoperatively. Lastly, our study findings indicate an association between minimally invasive surgery and decreased fused levels, blood loss, and comparable complications, but the small patient population and homogenous sample do not provide enough data to support a causal relationship.

## Conclusions

Surgical management of hyperostotic spine fractures of the thoracic/thoracolumbar spine can be performed either through the MIS-PPS or OSM. Both surgical techniques have equivalent capabilities in managing unstable spine fractures in the setting of hyperostotic spine disease. In the case of MIS-PPS, patients tend to have shorter operative times, fewer levels of instrumentation, and reduced intraoperative blood loss, compared to OSM. There was no significant difference between the two surgical techniques in developing postoperative infection, epidural hematoma, or implant revision.
